# Navigating Idiopathic Masseter Muscle Hypertrophy in a 14-Year-Old Female Child: A Report of a Unique Case

**DOI:** 10.7759/cureus.52792

**Published:** 2024-01-23

**Authors:** Chaitanya Kumar Javvaji, Keta Vagha, Jayant D Vagha, Rahul Desale, Punam Uke, Ashish Varma, Anirudh Kommareddy, SreeHarsha Damam, Naramreddy sudheesh Reddy

**Affiliations:** 1 Pediatrics, Jawaharlal Nehru Medical College, Datta Meghe Institute of Higher Education and Research, Wardha, IND; 2 Radiology, Jawaharlal Nehru Medical College, Datta Meghe Institute of Higher Education and Research, Wardha, IND

**Keywords:** sialography, benign vascular tumors, mandible tumor, botulinum injection, masseter muscle, parotid tumours

## Abstract

This case report documents the clinical journey of a 14-year-old female child experiencing bilateral swelling and pain in the mandibular region, suggestive of idiopathic masseter muscle hypertrophy. This condition, although relatively uncommon, can present itself either unilaterally or bilaterally. While cosmetic concerns, often denoted as a “square face,” are commonly expressed by patients, additional symptoms like protrusion, bruxism, or trismus may also be present. The patient reported a gradual and asymptomatic bilateral bulging in the mandible angle region, with an explicit complaint of pain. The physical examination revealed bilateral masseter hypertrophy without accompanying local inflammatory changes. Diagnosing this condition necessitates discerning its characteristics, evaluating clinical and radiographic findings, and excluding more severe pathologies like parotid diseases, lymphangioma, and rhabdomyoma. In cases of diagnostic uncertainty, complementary tests are deemed appropriate. Treatment strategies range from conservative measures to surgical interventions. This investigation aims to fulfill its primary objectives by presenting a case study elucidating the intricacies of idiopathic masseter hypertrophy, detailing associated symptoms, and exploring the spectrum of potential treatment options. Through this exploration, we contribute to the evolving understanding and management of this unique condition, especially within the pediatric age group.

## Introduction

The masseter muscle’s pivotal role in effective mastication and its lateral location near the mandibular ramus underscore its significance in facial aesthetics. Idiopathic masseter muscle hypertrophy (IMMH), first reported by Legg in 1880, delineates a distinct clinical presentation typified by anomalous enlargement of the masseter muscle [1]. Despite an array of cited factors such as clenching, malocclusion, temporomandibular joint disorders, or bruxism, a definitive causal link to IMMH remains elusive. This condition carries significant implications for both functional and aesthetic aspects, as an enlarged masseter muscle can influence facial contours, causing discomfort and adverse cosmetic effects for many individuals [2].

Patients frequently seek medical consultation motivated by aesthetic considerations, particularly when the hypertrophy is one sided, resulting in conspicuous asymmetry in the lower one-third of the face [3]. The peak occurrence of IMMH is typically noted in individuals during their second and third decades of life, without discernible gender predilection [4]. While there is a congenital variant, acquired masseter hypertrophy is more prevalent, typically presenting bilaterally and symmetrically. Nonetheless, asymmetry is not uncommon, especially in cases where patients demonstrate a preference for chewing or clenching on one side [5]. In this case report, we delve into a detailed exploration of a specific instance of IMMH, examining its clinical intricacies, diagnostic challenges, and the application of innovative treatment modalities. Through this investigation, we aim to contribute valuable insights into the evolving landscape of IMMH management.

## Case presentation

**Figure 1:**
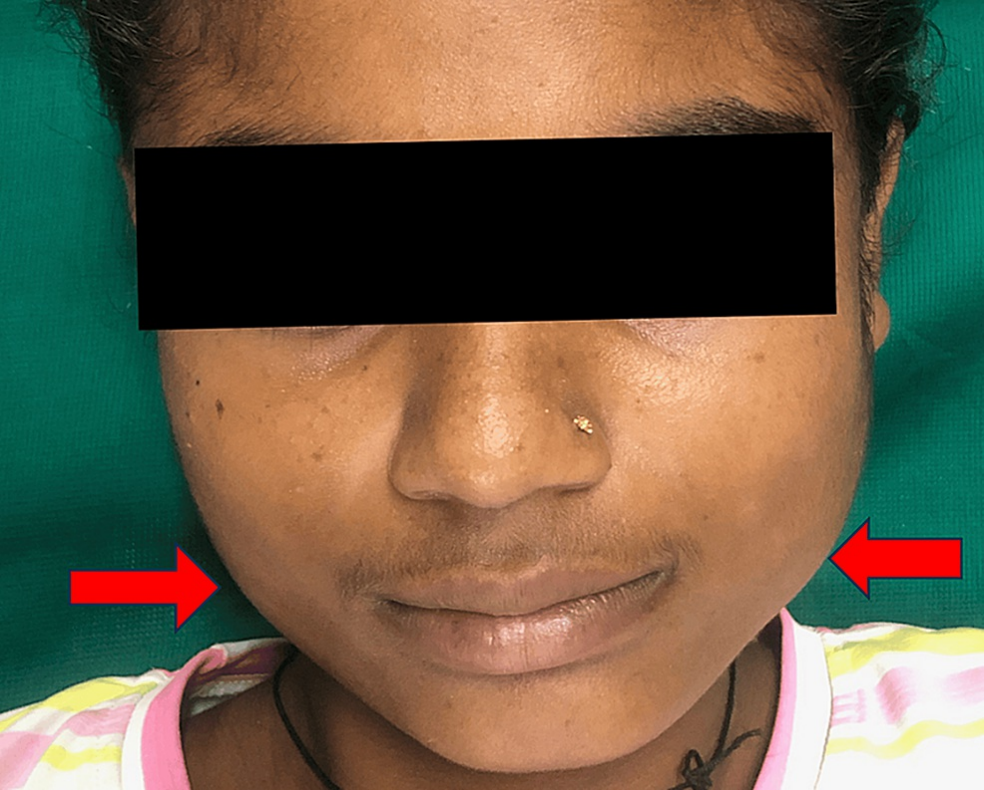
Clinical image showing bilateral mandibular prominance

A 14-year-old female was brought to Acharya Vinoba Bhave Rural Hospital by her parents through a medical camp, reporting complaints of bilateral swelling and pain in the mandibular region that persisted for 1.5 years. As per parental narration, the child was asymptomatic for 1.5 years prior but subsequently developed insidiously onset, gradually progressive bilateral mandibular swellings with no identifiable aggravating or relieving factors. Clinical examination disclosed a soft tissue mass located proximal to the angle of both the right and left mandibular bodies (Figure 1), exhibiting heightened prominence during jaw clenching. Mandibular movements were observed to be within normal limits. Upon physical examination, bilateral masseter muscle bulging was evident, and an extraoral assessment highlighted a notable bilateral swelling centered over the mandibular angle. The palpation of the swelling indicated a normal tissue tone and the absence of tenderness. Importantly, there was an absence of reported temporomandibular joint clicking, facial trauma, dental abnormalities, or any familial history indicative of masseter hypertrophy. Furthermore, there was no documented evidence of systemic diseases in the patient’s medical history.

**Figure 2:**
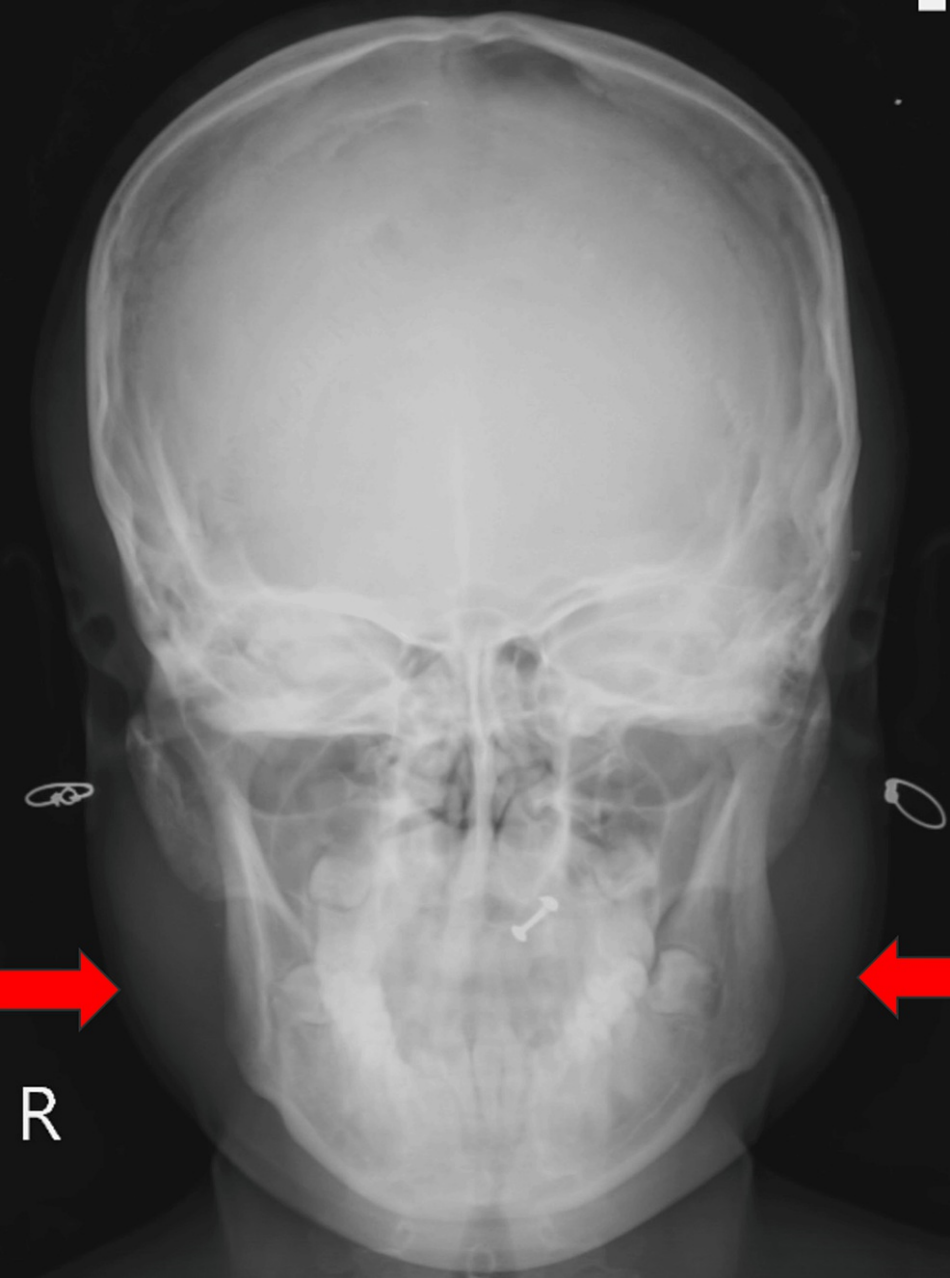
Mandible X-ray

Anteroposterior radiography showed swelling over both sides of the mandible (Figure 2).

**Figure 3:**
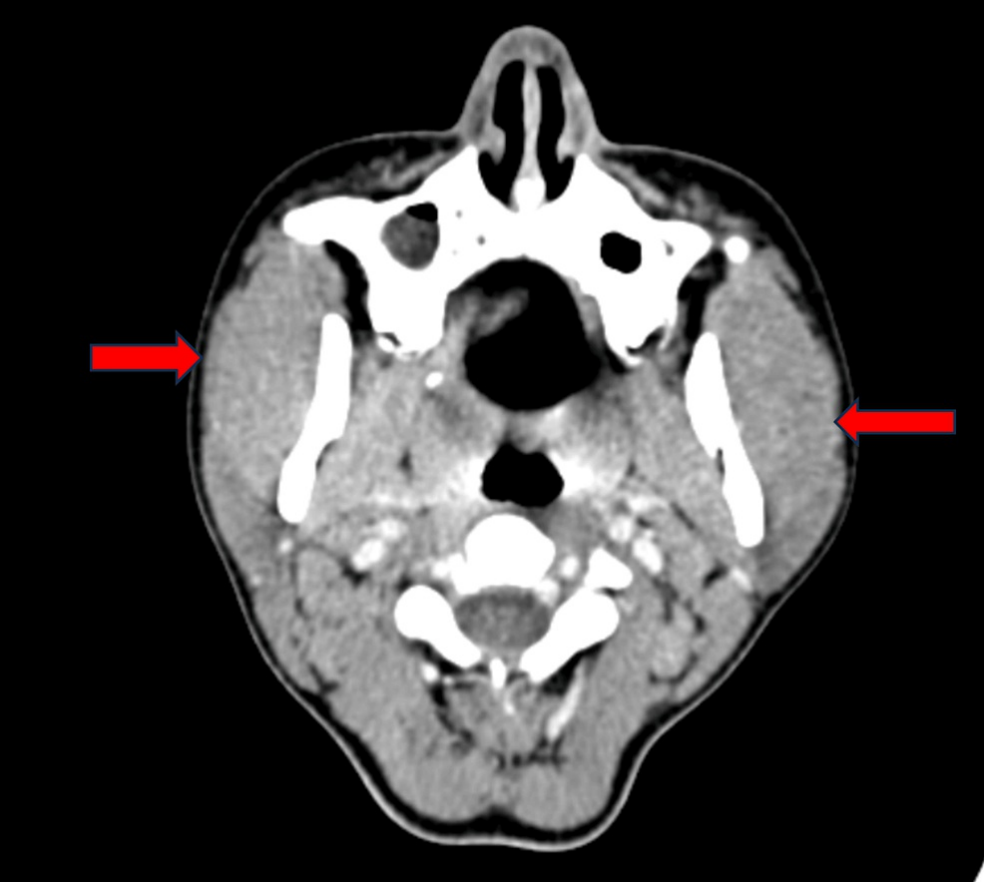
CT scan axial view showing bulky masseter muscle hypertrophy (red arrows)

**Figure 4:**
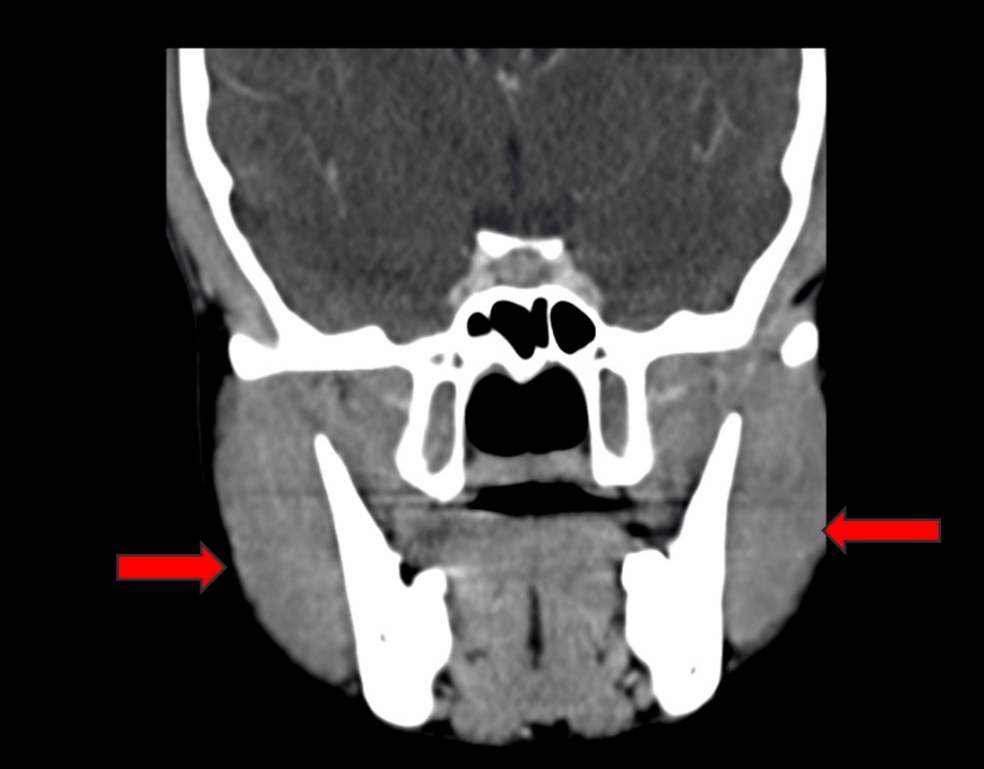
CT scan coronal view showing bilateral masseter muscle hypertrophy (red arrows)

An oro-maxillofacial surgery consultation was initiated, resulting in the recommendation for a CT for further evaluation. The CT findings indicated bilateral masseter muscle hypertrophy without discernible focal lesions in axial and coronal views (Figures 3, 4). A subsequent review of the oro-maxillofacial surgery consultation advised symptomatic management.

In this case, the child experienced relief from symptoms following the prescription of analgesics and engagement in physical therapy. The child’s parents were extensively counseled regarding the condition and presented with comprehensive treatment options, encompassing both surgical and medical management, including the consideration of botulinum toxin. Although a one-month follow-up was recommended, regrettably, the patient did not return for subsequent assessments. Efforts to re-establish contact for subsequent evaluation were undertaken. Written consent was obtained, authorizing the disclosure of images in the case report.

## Discussion

The masseter is a robust quadrilateral masticatory muscle. Masseter hypertrophy appears as a persistent, asymptomatic enlargement of either unilateral or bilateral masseter muscles, primarily induced by hypertrophy from activities like bruxism or clenching. This condition is prevalent in younger individuals. In advanced age groups experiencing dental deterioration, the diminished ability to fully activate the masseters often leads to the regression of pre-existing masseter hypertrophy. From an anatomical perspective, the heightened thickness of the masseter is focused along the inferior part of the mandibular ramus, causing a distinctive alteration in the usual facial contour to a rectangular configuration [6]. IMMH is deemed a rare condition, notwithstanding the increasing aesthetic concerns voiced by patients. It exhibits a higher prevalence among individuals of Asian descent. Baek et al. conducted an in-depth analysis, examining 108 documented cases until 1984, unveiling significant demographic insights. The average age of the patients was 30 years, with 57% being males. Bilateral involvement was observed in 60% of cases, while only five cases demonstrated concurrent temporalis muscle hypertrophy [7].

Bone spurs at the mandibular angle are frequently identified in cases of masseter muscle hypertrophy. However, it is important to note that Bloem and van Hoof [8] highlighted that approximately 20% of the general population may display this characteristic. Therefore, it cannot be definitively considered a diagnostic indicator. Diagnosing masseter muscle hypertrophy necessitates a comprehensive approach, incorporating clinical examination, targeted interviews, X-rays, and muscle palpation. In the current case, the enlargement of the masseter muscle was discerned during the physical examination.

CT serves as a well-established diagnostic modality, offering intricate details about anatomical landmarks and adjacent structures. Its indispensability becomes evident in masseter muscle hypertrophy with bone flaring, owing to its high-resolution imaging capabilities for bone structures and direct visualization. This attribute is not attainable with MRI, as the cortical bone fails to generate a significant signal [9]. In the current case report, CT revealed bilateral hypertrophy of the masseter muscles.

The differential diagnosis for the presented case included parotid tumors, lipoma, parotiditis, mandible tumors, vascular tumors, and muscle tumors. In unilateral cases, achieving an accurate diagnosis can be challenging, necessitating careful differentiation from parotid gland alterations. In such instances, performing a sialography is essential to rule out this possibility [10]. Clinical management involves a multifaceted approach. This encompasses counseling for individuals with underlying psychiatric disorders, analgesics, physical therapy, and the administration of antispasmodic and anxiolytic drugs. Favorable outcomes are often observed in patients with mild hypertrophy. Nevertheless, there is a scarcity of dependable reports in the existing literature regarding the success rates of standalone clinical therapy. Thus, a comprehensive and individualized treatment plan is imperative for optimal patient care.

In 1947, Gurney pioneered the concept of surgical intervention for masseter muscle hypertrophy, introducing a procedure that involves making a submandibular incision, through which three-fourths to two-thirds of the accessible muscle tissue is excised [11]. The extraoral approach is frequently utilized for addressing IMMH. The procedure is performed via a submandibular incision, followed by the excision of the masseter muscle to two-thirds of its thickness. An alternative treatment option involves administering botulinum toxin type A into the masseter muscle. This method is reported as a safe and effective modality for various conditions, including sialorrhea, orofacial dystonia, muscle hypertrophy, and Frey's syndrome. Botulinum toxin type A is derived from *Clostridium botulinum*. Its introduction into the muscle disrupts the neurotransmitter mechanism, leading to targeted paralysis and subsequent muscle atrophy [12-14].

In the presented case, the child’s symptoms were alleviated through the administration of analgesics and physical therapy. Despite being advised for a follow-up after one month, the patient, unfortunately, was lost to follow-up.

## Conclusions

The case of masseter hypertrophy underscores the importance of employing a comprehensive diagnostic approach and emphasizes the necessity of contemplating a diverse array of potential differential diagnoses. Masseter hypertrophy should be considered in patients exhibiting facial asymmetry, mandibular enlargement, or functional concerns, particularly in the presence of a history of bruxism or persistent cosmetic worries. Furthermore, the challenges in managing masseter hypertrophy underscore the need for tailored and individualized treatment plans. Overall, this case report emphasizes the importance of a thorough diagnostic process and advocates for a multidisciplinary approach to enhance patient care and achieve favorable outcomes in instances of masseter hypertrophy.
